# NMR-based metabolomics in Alzheimer’s disease research: a review

**DOI:** 10.3389/fmolb.2023.1308500

**Published:** 2023-11-30

**Authors:** Alessia Vignoli, Leonardo Tenori

**Affiliations:** ^1^ Department of Chemistry “Ugo Schiff”, University of Florence, Sesto Fiorentino, Italy; ^2^ Magnetic Resonance Center (CERM), University of Florence, Sesto Fiorentino, Italy; ^3^ Consorzio Interuniversitario Risonanze Magnetiche MetalloProteine (CIRMMP), Sesto Fiorentino, Italy

**Keywords:** NMR, metabolites, lipoproteins, Alzheimer’s disease, mild cognitive impairment

## Abstract

Alzheimer’s disease (AD) is a progressive neurodegenerative disorder and represents the most common cause of dementia in the elderly population worldwide. Currently, there is no cure for AD, and the continuous increase in the number of susceptible individuals poses one of the most significant emerging threats to public health. However, the molecular pathways involved in the onset and progression of AD are not fully understood. This information is crucial for developing less invasive diagnostic instruments and discovering novel potential therapeutic targets. Metabolomics studies the complete ensemble of endogenous and exogenous metabolites present in biological specimens and may provide an interesting approach to identify alterations in multiple biochemical processes associated with AD onset and evolution. In this mini review, we summarize the results from metabolomic studies conducted using nuclear magnetic resonance (NMR) spectroscopy on human biological samples (blood derivatives, cerebrospinal fluid, urine, saliva, and tissues) from AD patients. We describe the metabolic alterations identified in AD patients compared to controls and to patients diagnosed with mild cognitive impairment (MCI). Moreover, we discuss the challenges and issues associated with the application of NMR-based metabolomics in the context of AD research.

## 1 Introduction

Alzheimer’s disease (AD) represents an irreversible neurodegenerative condition characterized by a gradual deterioration of memory, cognitive abilities, and eventually the capacity to perform basic daily tasks. It stands as the predominant neurodegenerative ailment in the elderly population, impacting approximately 5%–7% of individuals aged 60 and above (Foley et al., 2017). In the clinical practice, the identification of AD-affected individuals is facilitated by measuring the levels of cerebrospinal fluid (CSF) core AD biomarkers, namely total tau (t-tau), threonine-181-phosphorylated-tau (p-tau) proteins, and amyloid beta 1–42 peptide (Aβ42) (Blennow et al., 2010). The variation in concentration of these molecules reflects the key aspects of disease pathogenesis (i.e., neuronal degeneration, tangles formation, and aggregation and deposition of amyloid plaques). During the asymptomatic phase of AD, CSF analysis in affected individuals commonly reveals diminished concentrations of Aβ42, and elevated levels of t-tau and p-tau proteins (Mattsson et al., 2009). Remarkably, these characteristic alterations are evident prior to the onset of clinical symptoms. Thus, the identification of AD-affected individuals is facilitated by the detection of abnormal levels of these CSF core biomarkers, even in the prodromal phase (Dubois et al., 2014).

Despite the clinical utility of these biomarkers, the intricate molecular pathways contributing to the onset and progression of AD remain incompletely elucidated. The imperative to uncover novel molecular targets for AD, with applications in early diagnosis, prognosis, disease trajectory prediction, and therapeutic interventions, underscores the critical need for comprehensive insights into the underlying molecular biochemistry of AD (Paglia et al., 2016).

Metabolomics, a discipline dedicated to the identification, quantification, and characterization of the entire spectrum of endogenous and exogenous metabolites in a biological specimen, emerges as a promising avenue of exploration (Ashrafian et al., 2021). Metabolites, representing the downstream products of the genome, transcriptome, and proteome, as well as the upstream inputs from diverse external factors such as environment, lifestyle, diet, and drug exposure, encapsulate a holistic view of the biochemical landscape (Nicholson and Lindon, 2008).

Given these considerations, metabolomics presents itself as a compelling approach for investigating alterations in multiple biochemical networks throughout the course of AD. This approach holds potential not only for enhancing our understanding of the disease mechanisms but also for paving the way towards the identification of new, effective, and minimally invasive targets for early detection, prognosis, and therapeutic intervention in Alzheimer’s disease.

Mass spectrometry and Nuclear Magnetic Resonance (NMR) spectroscopy are the two main analytical platforms available to perform metabolomic analysis. MS overshadows NMR in terms of sensitivity, having a detection limit in the rage of nano-to picomolar concentrations, which translates in being able to quantify a number of compounds of the order of 10^3^. In contrast, NMR struggles to detect metabolites at concentrations below the micromolar level. On the other hand, NMR is intrinsically quantitative, high-throughput and highly reproducible on a wide dynamic range (Vignoli et al., 2019). To be exhaustive, NMR, performed at an intermediate field such as a 600 MHz (the metabolomics gold standard) and in complex samples with crowded spectra such as biofluids, is limited also by spectral resolution. Considering all the abovementioned aspects, NMR and MS can be considered complementary since the weaknesses of one platform can be compensated by the strengths of the other, and both can contribute to AD research (González-Domínguez et al., 2017). Our review is focused on NMR which in the last years has demonstrated to be a powerful tool for searching novel biomarkers (Emwas et al., 2019) for disease diagnosis, prognosis, monitoring patients during therapeutic treatments and finding novel potential therapeutic targets (Wishart, 2016; Holmes et al., 2018; McCartney et al., 2019; Vignoli et al., 2020b; Jobard et al., 2021; Buergel et al., 2022; Vignoli et al., 2022).

In this review we decided to collect the main finding obtained from metabolomic studies performed using NMR spectroscopy on human biological samples from AD patients. Providing a comprehensive and in-depth methodological description of the use of NMR technique for metabolomic analyses is beyond the scope of this work. However, we refer interested readers to a recently published review by our group that specifically addresses these aspects for both liquid and semi-solid samples (Ghini et al., 2023).

## 2 Article selection

### 2.1 Study inclusion and exclusion criteria

The detailed study selection criteria are presented according to the Population, Exposure, Comparison, Outcome and Study design (PECOS) criteria as outlined below:

Inclusion criteria

⁃ P (Participants): Adult patients (>18 years of age) from any geographic location, any age or gender.
⁃ E (Exposure): Patients with confirmed diagnosis of AD.
⁃ C (Comparison): Difference in concentration of metabolites and lipoproteins between AD and other control/pathological groups.
⁃ O (Outcome): Dysregulation of metabolite/lipoproteins concentrations between the study groups.
⁃ S (Study Design): Human-based observational studies (case-control, cohort, or cross-sectional) that performed metabolomics via NMR to quantify the concentrations of metabolites and lipoproteins.

Exclusion criteria

⁃ Targeted metabolomic experiments that are used to validate and translate already identified metabolites from hypothesis generating studies.
⁃ Animal or cell-based studies.
⁃ Non-Observational study designs such as case reports, conference proceedings, letters to editor, reviews, and meta-analysis.
⁃ Metabolites quantified using analytical platforms other than NMR (e.g., mass spectrometry).
⁃ Studies published after October 2023.

### 2.2 Search and selection strategy

The search was conducted on the Web of Science electronic database in the title (TI) and in the topic (TS) fields, using a combination of keywords paired with the Boolean operator “AND”. The final query was (TI=(alzheimer) AND TS = ((metabolomic* OR metabolite*) AND *NMR*)). The search was conducted the 16th of November 2023.

Applying the abovementioned inclusion and exclusion criteria, study selection was carried out screening titles and abstracts of all publications. Studies that matched the inclusion criteria but had insufficient details in abstracts were further examined by inspecting the complete texts.

**FIGURE 1:**
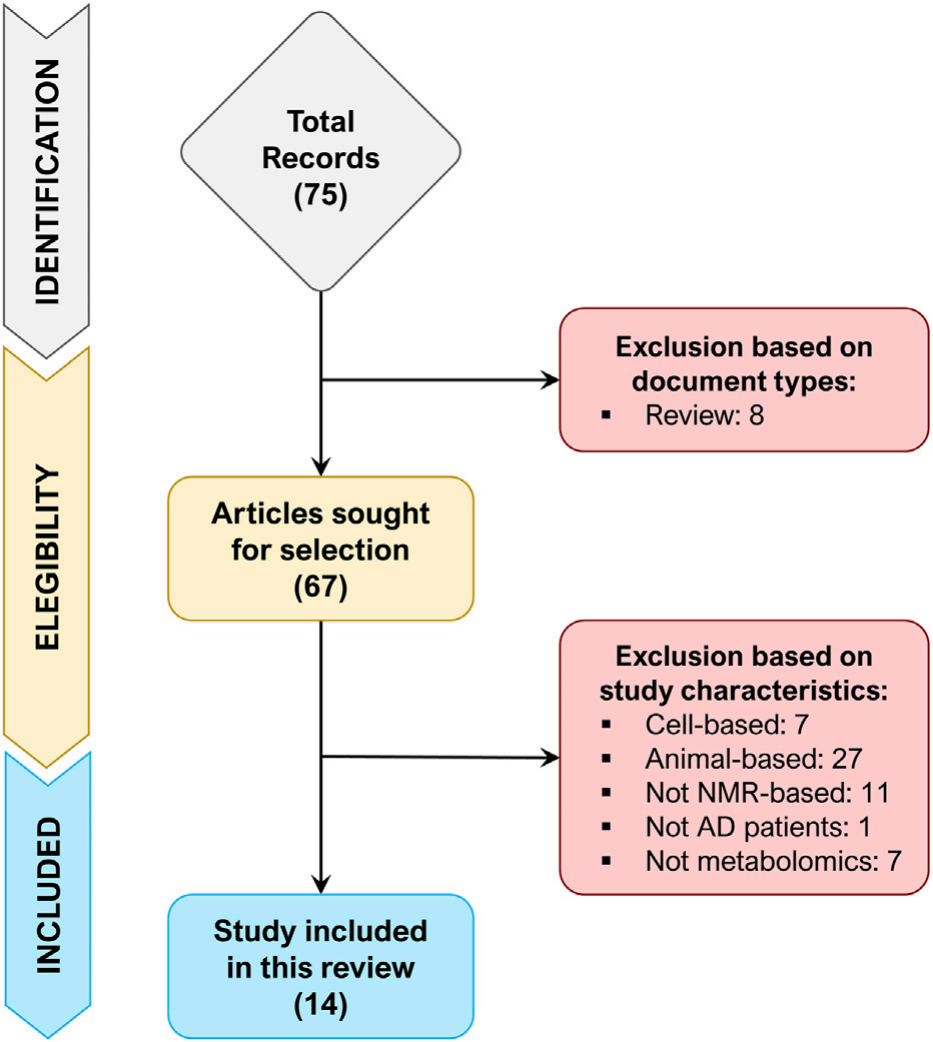
Flowchart of study identification, eligibility, and inclusion.

A total of 75 records were retrieved after the identification phase, out of which 8 records were excluded because of document type (review). The remaining 67 records were screened for their title and abstract (or entire text when required), of which 53 records were excluded since did not match the inclusion/exclusion criteria. Figure 1 depicts the flowchart of study selection, summarizing the process of study identification, eligibility, and inclusion.

## 3 Study characteristics

The characteristics of the 14 included studies are presented in Table 1. The selected studies were published in the last 11 years (2012–2023) and were mainly conducted in Europe and United States (86%). Of the studies that reported age and sex, the mean age of the enrolled population ranges from 54 to 82 years, with 47% of them being male. Most of the research compared metabolomics data between AD and healthy controls and MCI. The majority of the studies (7 out of 14) analyzed blood derivatives (plasma or serum), followed by cerebrospinal fluid (CSF), urine, tissues and saliva. Of the studies that reported the information, all samples were stored at −80°C pending NMR analysis as *per* the best practice for metabolomics, and, except for two studies, all samples were acquired using a spectrometer operating at 600 MHz.

**TABLE 1:** Detailed characteristics of the included studies.

Author/Year	Cohort allocation	Sample type	Cases/Controls	Age (mean)	Sex (male/female)	Sample storage	NMR (MHz)	Significant metabolites (AD as References)	Study limitations
Botosoa et al. (2012)	France	Tissue Extracts	AD 8 ALS 11	-	-	−80°C	500 (s)	AD vs. ALS ↑Ala, acetate, Glu, Gln, GPC ↓lactate, NAA, creatine, PC, choline, myo-inositol	- Very limited numerosity
									- Missing relevant demographic information
									- Results not independently validated
Graham et al. (2014)	England	Tissue Extracts	CTR 15 AD 15	82	CTR 4/11 AD 9/6	-	400 (s)	AD vs. CTR ↑Ala, taurine	- Very limited numerosity
									- Missing information on storage conditions
									- Statistical methods poorly described
									- Results not independently validated
									- Gender unbalance
Kim et al. (2017)	South Korea	Plasma	CTR 11 AD 9	78	CTR 2/7 AD 1/10	-	600 (s)	AD vs. CTR ↑Glu, Gln, Leu, oxaloacetate, Asp, Ile, 3-hydroxyisovalerate	- Very limited numerosity
									- Missing information on storage conditions
									- Statistical methods poorly described
									- Gender unbalance
									- Results not independently validated
									- Fasting status not reported
Vignoli et al. (2020a)	Netherlands	CSF	nonAD 20 MCI-AD 20 AD 20	54	nonAD 14/6 MCI-AD 12/8 AD 6/14	−80°C	600 (s)	AD vs. nonAD ↓Val, acetate, 3-hydroxyisovalerate AD vs. MCI-AD ↓Val, 3-hydroxyisovalerate	- Limited numerosity
	Italy	CSF	nonAD 12 AD 14	60	nonAD 6/6 AD 6/8				- Gender unbalance
									- Results reproduced in 2 cohort but not independently validated
Yilmaz et al. (2017)	Michigan	Saliva	CTR 12 MCI 8 AD 9	83	CTR 4/8 MCI 3/5 AD 3/6	−80°C	600	AD vs. CTR^#^ ↑Propionate, Acetone AD vs. MCI ↑Creatinine, 5-aminopentanoate	- Very limited numerosity
									- Statistical methods poorly described
									- Results not independently validated
Zhang et al. (2019)	Georgia	Intact Tissue	CTR 11 AD 11	63	CTR 7/4 AD 6/5	−80°C	600 (HR-MAS)	AD vs. CTR ↑PC/Creatine, GPC/Creatine, α&β-Glc/Creatine ↓NAA/Creatine, Ace/Creatine, GABA/Creatine, Asp/Creatine, Myo-Inositol/Creatine, Taurine/Creatine	- Very limited numerosity
									- Results not independently validated
Yilmaz et al. (2020a)	Michigan	Urine	CTR 29 MCI 10 AD 20	79	CTR 13/16 MCI 5/5 AD 9/11	−80°C	600 (s)	AD vs. CTR	- Limited numerosity
								↑2-Hydroxybutyric acid, Trimethylamine, Trimethylamine-n-oxide, Pro ↓2-Hydroxyisovaleric acid, Alpha-ketoisovaleric acid, D-Glucose, Pyridoxine, Glycolic acid	- Results not independently validated
								AD vs. MCI	
								↑2-Hydroxybutyric acid, 3-Hydroxyisovaleric acid, 5-Aminopentanoic acid, Cytosine, D-Glucose, Guanidoacetic acid, Hippuric acid, Ala, Myo-inositol	
								↓2-Hydroxyisovaleric acid, Alpha-ketoisovaleric acid, Dimethylsulfone, Mannitol, Methanol, Trimethylamine, Tryptophan, Ile, Acetate, Acetone	
Yilmaz et al. (2020b)	Michigan	Plasma	CTR 101 MCI 71 AD 77	79	CTR 48/53 MCI 37/34 AD 34/43	−80°C	600 (s)	AD vs. CTR	- Blood components were separated within 24 h
								↑Glycerol, Gln, Val	- Results not independently validated
								AD vs. MCI	
								↑Arg, Creatinine, Sarcosine	
								↓Acetate, Acetoacetate, Carnitine, Isopropyl alcohol, Acetone, Dimethyl sulfone	
Kurbatova et al. (2020)	Europe	Urine	CTR 214 stable MCI 200 dementia MCI 55 AD 197	77	CTR 103/111 stable MCI 99/101 dementia MCI 20/35 AD 101/96	−80°C	600 (s)	AD vs. CTR	- Results not independently validated
								↑Sucrose	
Di Costanzo et al. (2020)	Italy	Serum	CTR 51 SMD 40 MCI 40 AD 40	68	CTR 26/25 SMD 14/26 MCI 14/26 AD 13/27	−80°C	600 (s)	AD vs. CTR	- Results not independently validated
								↑Gln	
								↓Acetate, Choline, Ile, Leu, Val	
								AD vs. MCI	
								↑Glucose, Glyceryl lipids, Lactate	
								↓Acetate, Choline, Ile, Leu, Val	
								AD vs. SMD	
								↑Acetate, Choline, Methanol, PC/GPC	
								↓Ala, Ile, Leu, Val	
Weng et al. (2022)	Massachusetts	Plasma	CTR 19 AD 16	69	CTR 9/10 AD 9/7	−80°C	600 (HR-MAS)	AD vs. CTR	- Limited numerosity
								↑3-phosphoglycerate, fructose-6-phosphate, glucose-6-phosphate, myo-inositol, betaine, methyl-His, glycerylphosphorylcholine, ergothioneine, taurine, Gly, Ser, Trp	- Results not independently validated
								↓2-oxoglutarate, citrate, malate, glutathione disulfide, carnosine, ornithine, Ala, Gln, Val	
Berezhnoy et al. (2022)	Germany	Serum	CTR 54 MCI 51 AD 56	70	CTR 25/29 MCI 34/17 AD 31/25	−80°C	600 (s)	AD vs. CTR	- Results not independently validated
								↑LDL-2 cholesterol, LDL-3 cholesterol	- Samples collected at different fasting conditions
								AD vs. MCI	- Statistical methods poorly described
								↑VLDL-1 cholesterol, VLDL-1 triglycerides	
Berezhnoy et al. (2023)	Germany	CSF	CTR 20 MCI 22 AD 29	68	CTR 13/7 MCI 16/6 AD 14/15	−80°C	600 (s)	AD vs. CTR	- Limited numerosity
								↑Alpha-ketoisovaleric acid, 2-Hydroxy-isovalerate	- Results not independently validated
								↓2-hydroxybutyrate, 3-Hydroxy-isobutyrate	- Gender unbalance
								AD vs. MCI	- Statistical methods poorly described
								↑2-Hydroxy-isovalerate	
								↓Val, formate	
		Serum	CTR 29 MCI 21 AD 26	69	CTR 15/14 MCI 15/6 AD 13/13			AD vs. CTR	
								↓Glu, ornithine	
								AD vs. MCI	
								↓Glucose	
Botello-Marabotto et al. (2023)	Spain	Serum	CTR 50 MCI 27 AD 51	80	CTR 17/33 MCI 10/17 AD 9/42	−80°C	600 (s)	AD vs. CTR	- Results not independently validated
								↑Acetone, Ala, Creatine, Gly, Methanol, Lys, N-acetyled compounds, N-acetylglucosamine, Phe, Pyruvate, Thr	- Gender unbalance
								↓Acetylcholine, Choline, Ethanol, Ile, Glycerol	
								AD vs. MCI	
								↑Creatine, Glycine, Lactate, Lys, N-acetyled compounds, N-acetylglucosamine, Phe, Pyruvate	
								↓Choline, Ethanol, Glycerol	

CSF, cerebrospinal fluid; CTR, non-AD controls; MCI, mild cognitive impairment; AD, Alzheimer’s disease; SMD, subjective memory decline; ALS, amyloid lateral sclerosis; (s): in solution NMR; HR-MAS, high resolution magic angle spinning; GPC, glycerophosphocholine; NAA, N-acetyl aspartate; PC, phosphocholine; sFA, saturated fatty acids; uFA, unsaturated fatty acids; GABA, γ-aminobutyric acid; α&β-Glc: resonance found at 3.71 ppm.

## 4 Metabolomic/lipoproteomic differences between AD and controls

Although abnormal levels of the CSF core AD biomarkers enable the accurate identification of patients affected by AD, a full comprehension of the underlying molecular mechanisms involved in the onset and progression of this pathology is still distant. For this reason, most of the published studies aim to detect and quantify, in different biospecimens, AD-relevant metabolites and/or lipoproteins in AD patients as compared to cognitively normal individuals (CTR).

The first metabolomic studies via NMR date back to 2012–2014, and both examined *post-mortem* brain tissue extracts (Botosoa et al., 2012; Graham et al., 2014). Botosoa et al. analyzed frontal cortex extracts of samples collected *post-mortem* from AD patients and from a control group constituted by amyotrophic lateral sclerosis (ALS) patients. The AD metabolic signature was characterized by high levels of alanine, acetate, glutamate, glutamine and glycerophosphocholine (GPC), and low levels of lactate, creatine, N-AcetyAspartate (NAA), phosphocholine (PC), choline and myo-inositol (Table 1). In particular, NAA is a biomarker of neuronal integrity in the brain and its reduction reflects deficiency and neuronal dysfunction. Therefore, this metabolite could play a pivotal role in AD pathogenesis. Graham et al. analyzed extracts of *post-mortem* brain Brodmann 7 region samples. Multivariate statistical analysis shows a clear distinction between AD and CTR samples, and the most relevant metabolites in the discrimination were alanine and taurine (Table 1). In 2019 the first NMR-based metabolomic study conducted on intact *post-mortem* tissues obtained from frontal cortex was published (Zhang et al., 2019). The authors reported that high levels of PC, GPC and low levels of NAA, acetate, GABA, aspartate, myo-inositol and taurine are characteristic features of samples of AD patients as compared to CTR (Table 1).

Going from tissues to biofluids, NMR-based metabolomics of blood derivatives (plasma or serum) has shown the potential to distinguish patients with AD from CTR with optimal results (Kim et al., 2017; Yilmaz et al., 2020b; Di Costanzo et al., 2020; Berezhnoy et al., 2022, Berezhnoy et al., 2023; Weng et al., 2022; Botello-Marabotto et al., 2023). Among metabolites (which include amino acids, carbohydrates, lipids, choline-derived metabolites, keto acids, and fatty acids) and lipoproteins (fraction and subfractions) identified and quantified in the studies, several metabolites and 2 lipoprotein subfraction of LDL cholesterol (Table 1) were described as differentially abundant between AD and CTR. However, there is not a clear consensus on the directions (up or down) of dysregulation of these metabolic alterations and on their significance (Table 1). The high heterogeneity emerged could be ascribed to several factors: the small sample size of most of the studies, the lack of independent validation cohorts in the majority of the studies, relevant differences in sample collection, processing, and analytical method employed (e.g. not all blood samples were collected pre-prandially), and not adequately addressing of potential confounding risk factors (e.g. not all studies enrolled patients and controls age and sex matched). These factors could significantly impact metabolite concentrations and may be leading factors for inconsistencies of reported results.

Two studies (Yilmaz et al., 2020a; Kurbatova et al., 2020) investigated the metabolic phenotype of AD in urine (Table 1). Both studies proposed a combined approach using both ^1^H NMR and mass spectrometry and showed that the urine phenotype can discriminate AD and CTR with high discrimination accuracy. Yilmaz et al. reported that 2-hydroxybutyric acid, trimethylamine, trimethylamine-n-oxide, proline have higher concentrations in AD patients, whereas 2-hydroxyisovaleric acid, alpha-ketoisovaleric acid, D-glucose, pyridoxine and glycolic acid have lower concentrations. In this case, using available information, it was not possible to distinguish between the metabolites quantified through NMR and those quantified through mass spectrometry; whereas Kurbatova et al. clearly differentiated the quantification assay and the only significant difference emerged by NMR is the increasing of sucrose. One study searched for diagnostic biomarkers of AD in saliva samples (Yilmaz et al., 2017) identifying differences in the concentrations of 22 metabolites in AD and MCI as compared to CTR. Moreover, the authors built two distinct logistic regression models: one, based on creatinine and 5-aminopentanoate, able to discriminate AD from MCI with 0.900 sensitivity and 0.944 specificity, and another, based on propionate and acetone, which discriminates AD from CTR with 0.909 sensitivity and 0.842 specificity (Table 1).

Since brain directly transfers its metabolites into CSF, the latter most likely reflects the brain biochemistry, and thus CSF is obviously the biofluid of choice when it comes to studying neurological disorders. However, the invasiveness of the sample collection procedure and associated ethical issues (especially for control/healthy individuals) have resulted in only one available NMR-based metabolomic study examining AD and CTR (Table 1). In this study (Berezhnoy et al., 2023) it is showed that alpha-ketoisovaleric acid and 2-hydroxy-isovalerate are upregulated in the CSF of AD patients respect to CTR, whereas 2-hydroxybutyrate and 3-hydroxy-isobutyrate are downregulated. Moreover, they identified sex-specific metabolite alterations that underling once more how sex is a relevant confounding factor when one wants to perform metabolomics analyses (Vignoli et al., 2018; Bell et al., 2021; Costanzo et al., 2022).

## 5 Metabolomic/lipoproteomic differences between AD and MCI

The AD “continuum” starting from cognitively normal subjects, begins with subjective memory decline, progresses to Mild Cognitive Impairment (MCI) and eventually reaches AD (Jessen et al., 2014). However, MCI subjects may not evolve into dementia, indeed only 20%–40% of patients progresses to AD (Tahami Monfared et al., 2022). Understanding the mechanisms underlying this progression could contribute to addressing the still unsolved question of AD pathogenesis and evolution.

Two studies (Vignoli et al., 2020a; Berezhnoy et al., 2023) analyzed cerebrospinal fluid of AD and MCI patients by NMR, revealing a clear distinction between the two groups. Both studies consistently found a reduction in valine levels in the CSF of AD patients (Table 1). Furthermore, Vignoli et al. showed that valine is reduced even in comparison to MCI-AD patients, and that it correlates with patient cognitive decline. In addition to valine, Vignoli et al. also identified in AD patients a reduction in the levels of acetate and 3-hydroxyisovalerate. Conversely, Berezhnoy et al. reported higher CSF levels of 2-hydroxy-isovalerate and lower levels of formate in AD patients.

Focusing the attention from a compartmentalized biofluid such CSF to systemic biofluids, we were able to find five studies conducted on blood derivatives and two studies on urine (Table 1). Among the five studies on plasma/serum, four were focused on metabolite analysis (Yilmaz et al., 2020b; Di Costanzo et al., 2020; Berezhnoy et al., 2023; Botello-Marabotto et al., 2023) and reported 25 differentially abundant metabolites between AD and MCI. As observed in the comparison between AD and CTR, there is some inconsistency among the results (e.g. glucose, a metabolite which is very sensitive to sample collection and pre-analytical procedures, is reported reduced in AD in one study and increased in another study); however, two out of three studies described a significant reduction acetate in AD patients and it has been hypothesized that it could play a role in the compromission of the neurotransmission activity of acetylcholine (Di Costanzo et al., 2020).

Berezhnoy et al. focused their analysis on lipoproteins, using a commercially available quantitative lipoprotein assay based on 600 MHz NMR spectroscopy. They were able to correlate a set of 112 lipoprotein variables with clinical metadata and AD core biomarkers in CSF. They obtained a deeper insight into the pathophysiology of dementia and reported an increase of VLDL-1 cholesterol and VLDL-1 triglycerides in AD patients as compared to MCI (Berezhnoy et al., 2022). It is known that VLDL-1 cholesterol levels are correlated with ApoE4, that in turn is a factor that affect the Aβ levels: when a patient shows altered ApoE4 function, AD-related risks increase multiple-fold (Kloske and Wilcock, 2020). Further, the increase in VLDL-1 triglycerides in the AD group may be correlated with a prediabetic condition. Indeed, individuals with insulin resistance have a higher production of VLDL particles, which are responsible for triglyceride transport, reinforcing the idea of a close interconnection of blood lipoprotein biomarkers of type 2 diabetes and AD: some authors refer to AD as a type 3 diabetes (Accardi et al., 2012, 3). Putting things together, the authors speculated a strong link between VLDL parameters and amyloid plaque formation (Berezhnoy et al., 2022). This study provides a proof of concept that NMR-based lipoprotein analysis, in conjunction with metabolites analysis, is potentially able to provide an in-depth investigations of AD. This approach can be easily extended to further neurological diseases to provide interesting picture of the complex interplay among metabolites, lipoproteins, and clinical outcomes.

Among the two studies conducted on urine sample, only one reported the results of the comparison between AD and MCI (Yilmaz et al., 2020a), whereas in the other study MCI patients were enrolled to compare stable and dementia MCI (Kurbatova et al., 2020). Yilmaz et al. reported 19 urine metabolites significantly different between AD and MCI (Table 1). They used the concentrations of a panel of 10 metabolites (glucose, guanidinoacetate, urocanate, hippuric acid, cytosine, 2- and 3-hydroxyisovalerate, 2-ketoisovalerate, tryptophan, and malonate) to build a model able to discriminate the two groups with 78% sensitivity and 80% specificity. Based on these results, the authors suggests that urine metabolomics may be useful for developing a non-invasive test capable of diagnosing and distinguishing AD from MCI patients (Yilmaz et al., 2020a).

## 6 Conclusion

The NMR-based metabolomic studies presented in this review, despite their limitations, have demonstrated the existence of metabolic alterations in AD, albeit not consistent. Differences can be detected in brain tissue, blood serum/plasma, CSF, urine, and saliva. Moreover, there is evidence that some metabolic changes could predict the progression of AD. Therefore, NMR-based metabolomics could play a role in AD diagnosis and prognosis, serving as a valuable addition to classical clinical approaches.

NMR, with its ability to provide quantitative and extremely reproducible results, offers a valuable approach to understand the multifaceted and intricate metabolic landscape of AD. In particular, NMR-based metabolomics could play a role in: 1) long-term monitoring of AD evolution; 2) improving diagnosis and prognosis of AD on the base of their metabolic changes; 3) accelerate the discovery of metabolic biomarkers associated with AD; 4) characterize the biochemistry underlying AD with the final aim of identifying potential novel pharmaceutical targets. While this review is entirely focused on the NMR technique and its potential in AD research, we want to make it clear that we are neither implying that this is the sole analytical approach possible nor suggesting that this approach should replace others. On the contrary, the integration of NMR and MS in a multi-omics approach represents a powerful strategy, leveraging the strengths of both techniques. The synergy between these techniques in AD research extends beyond conventional boundaries, and holds promise for dissecting the complexities of the molecular mechanisms underlying neurodegeneration in AD.

Based on our review we foresee the need for improvement in enrolling larger and independent cohorts of patients and for a higher degree of standardization in the recommendations for sample collection, handling, preparation, acquisition, and data processing. These improvements would enable future researchers to obtain more robust, coherent, and interpretable results and facilitate the development of clinical applications for metabolomics.
